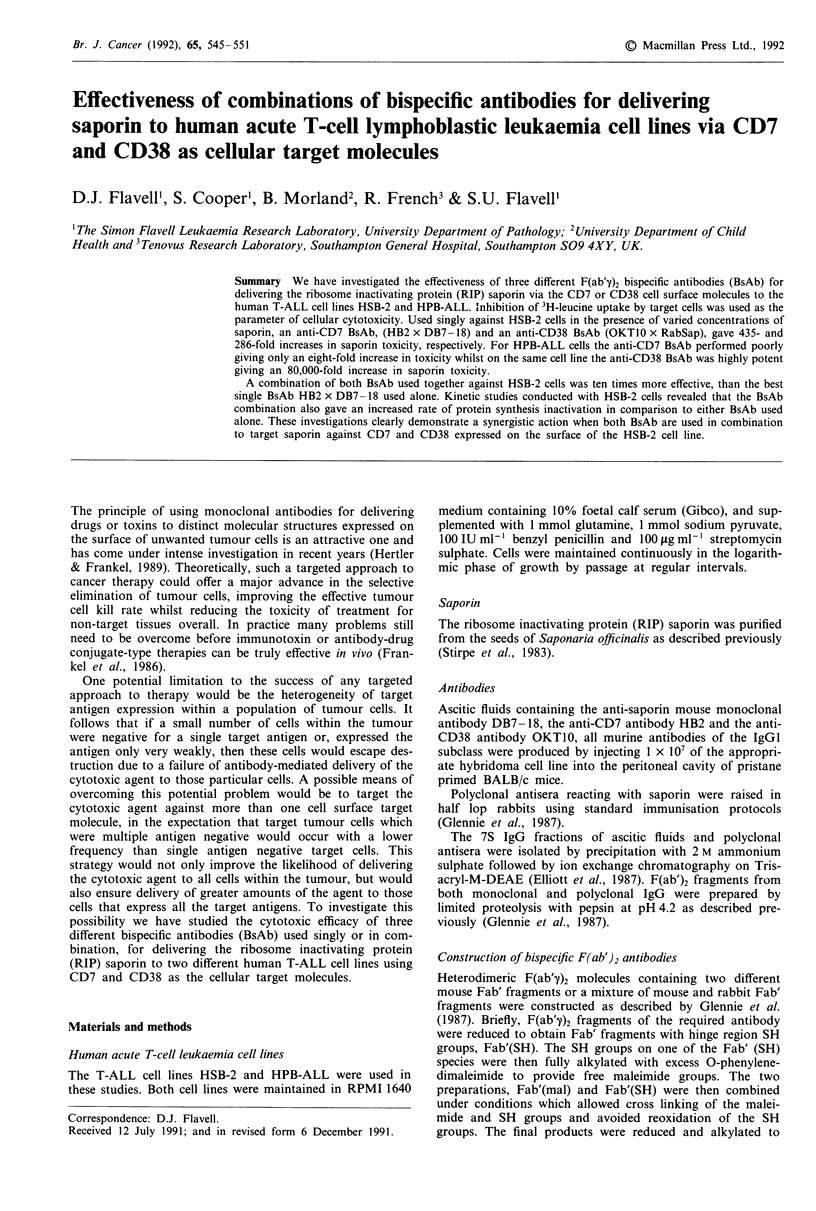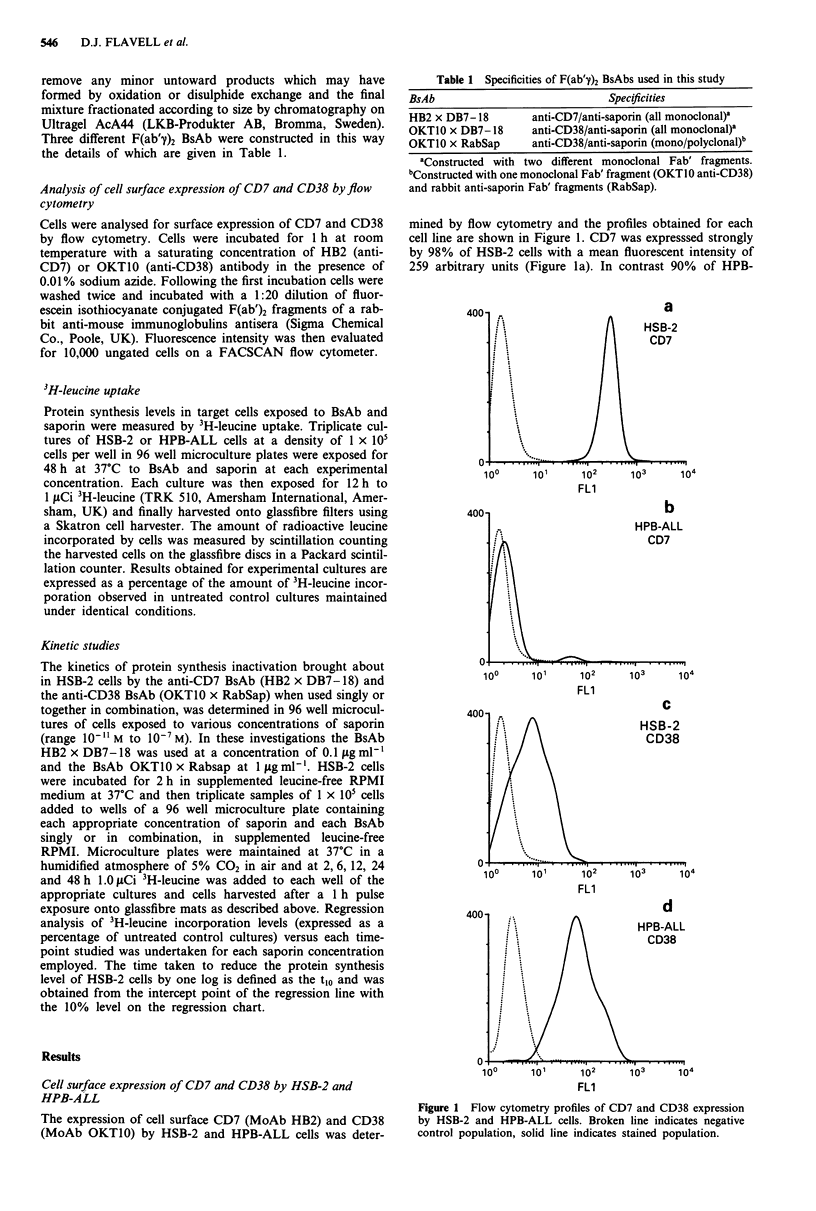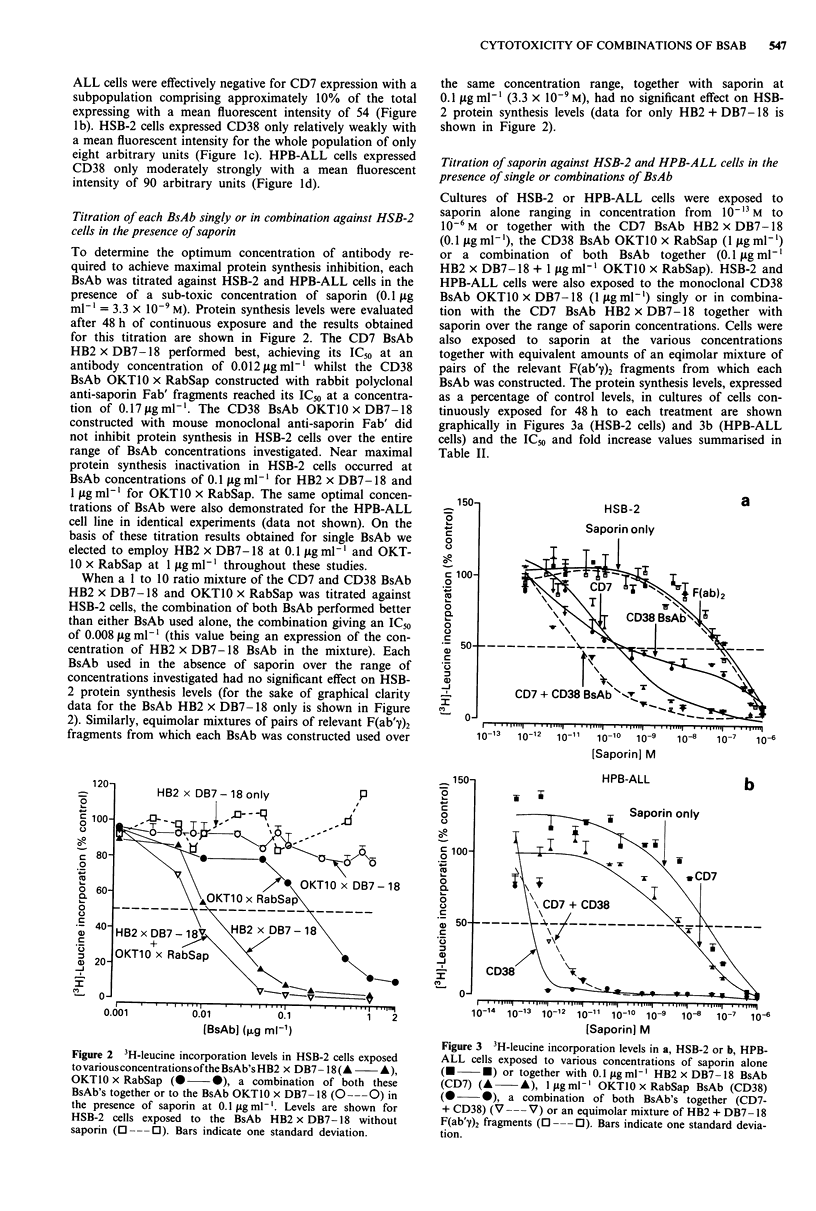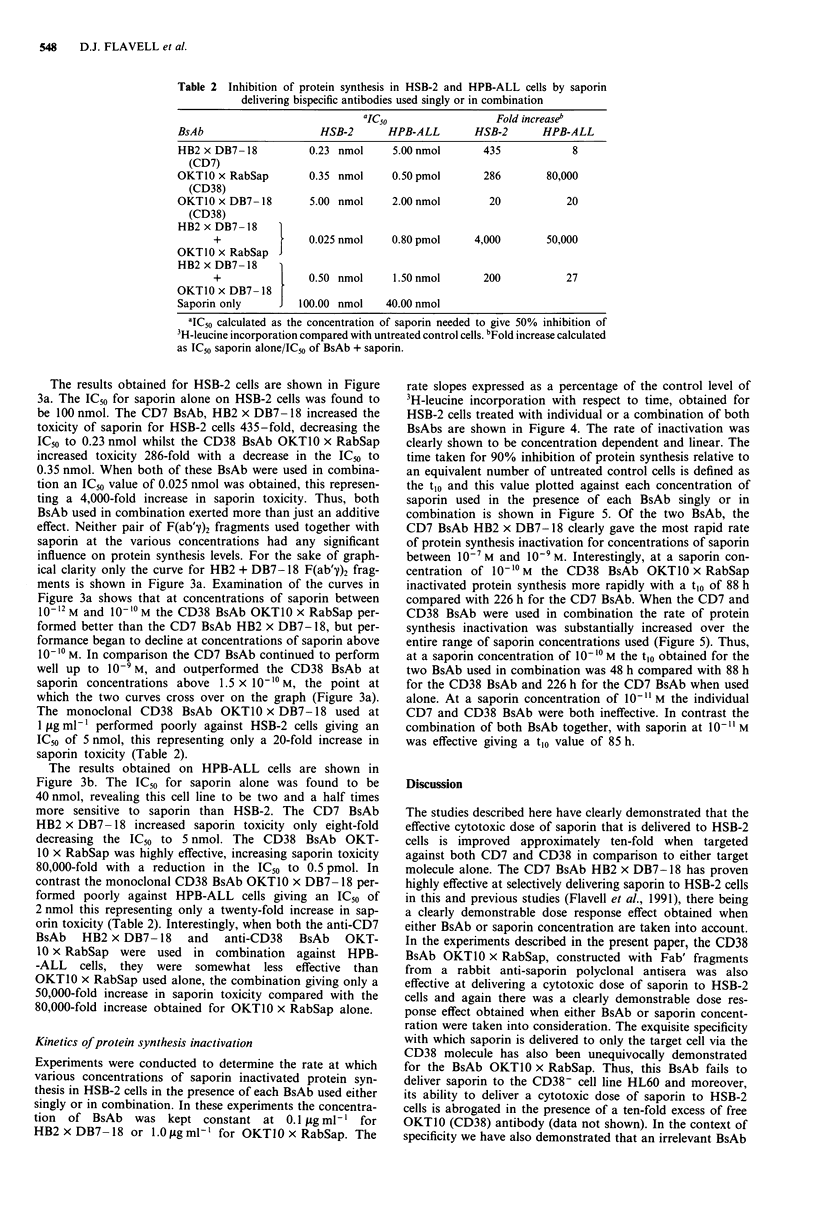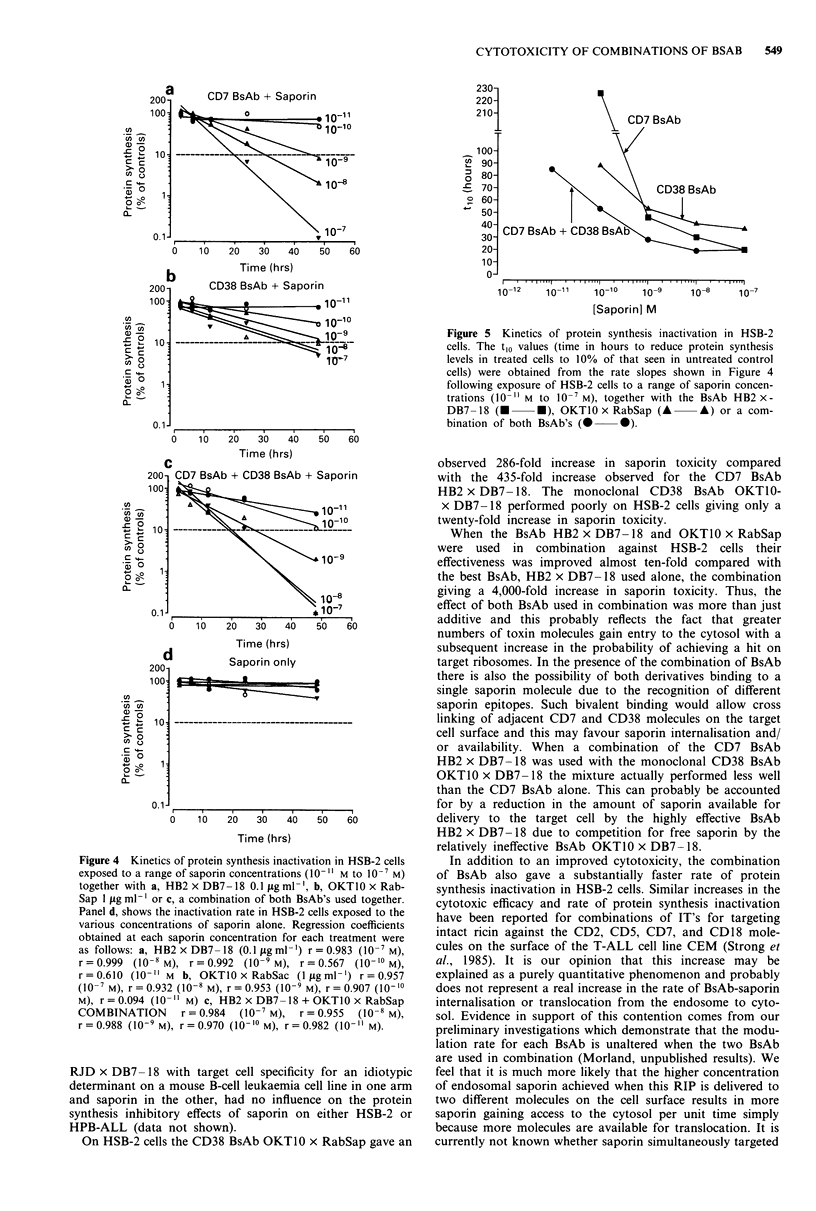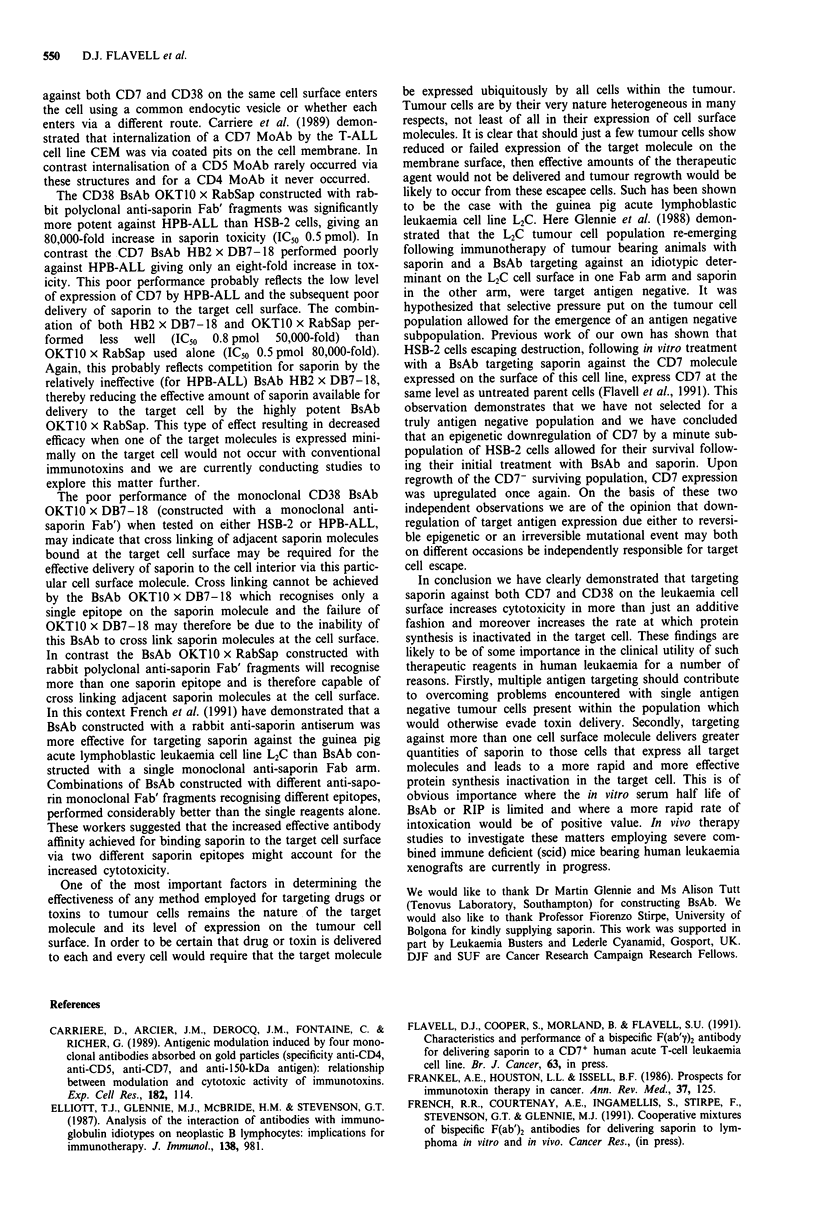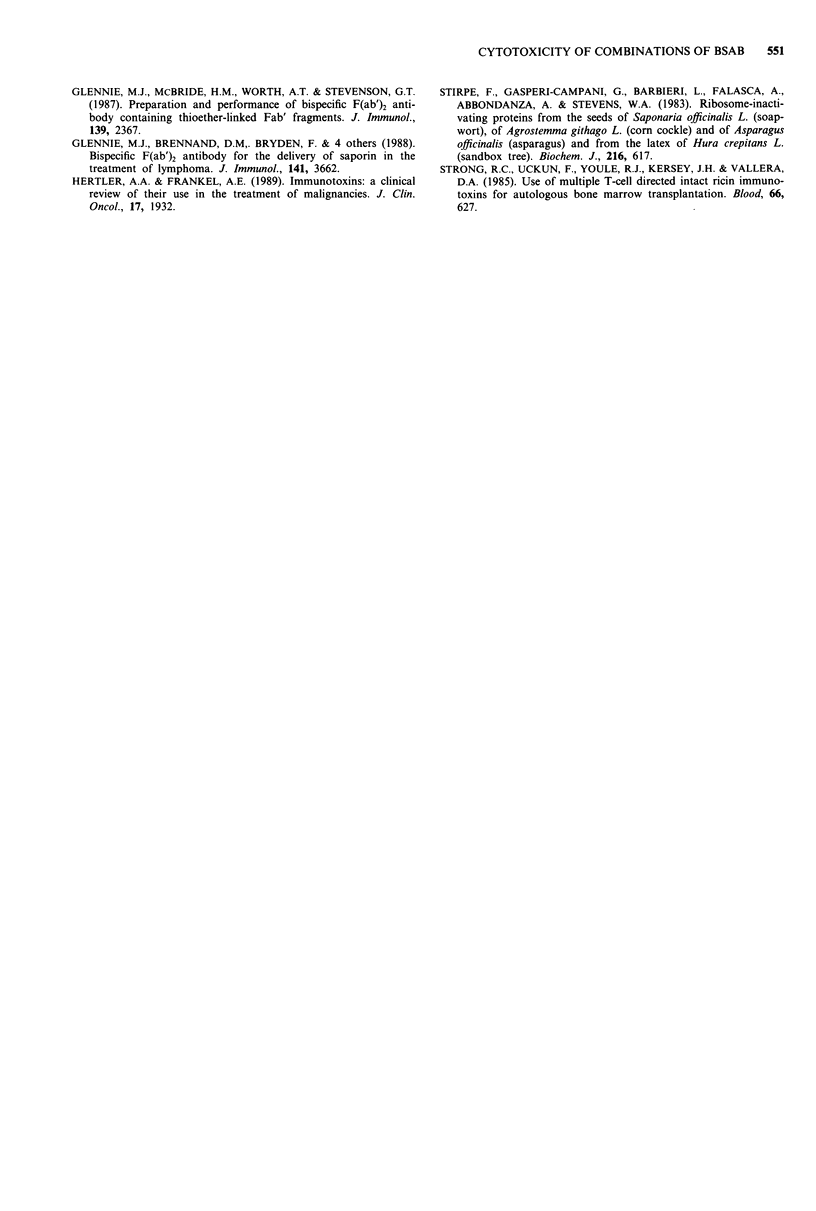# Effectiveness of combinations of bispecific antibodies for delivering saporin to human acute T-cell lymphoblastic leukaemia cell lines via CD7 and CD38 as cellular target molecules.

**DOI:** 10.1038/bjc.1992.112

**Published:** 1992-04

**Authors:** D. J. Flavell, S. Cooper, B. Morland, R. French, S. U. Flavell

**Affiliations:** Simon Flavell Leukaemia Research Laboratory, University Department of Pathology, Southampton, UK.

## Abstract

We have investigated the effectiveness of three different F(ab' gamma)2 bispecific antibodies (BsAb) for delivering the ribosome inactivating protein (RIP) saporin via the CD7 or CD38 cell surface molecules to the human T-ALL cell lines HSB-2 and HPB-ALL. Inhibition of 3H-leucine uptake by target cells was used as the parameter of cellular cytotoxicity. Used singly against HSB-2 cells in the presence of varied concentrations of saporin, an anti-CD7 BsAb, (HB2 x DB7-18) and an anti-CD38 BsAb (OKT10 x RabSap), gave 435- and 286-fold increases in saporin toxicity, respectively. For HPB-ALL cells the anti-CD7 BsAb performed poorly giving only an eight-fold increase in toxicity whilst on the same cell line the anti-CD38 BsAb was highly potent giving an 80,000-fold increase in saporin toxicity. A combination of both BsAb used together against HSB-2 cells was ten times more effective, than the best single BsAb HB2 x DB7-18 used alone. Kinetic studies conducted with HSB-2 cells revealed that the BsAb combination also gave an increased rate of protein synthesis inactivation in comparison to either BsAb used alone. These investigations clearly demonstrate a synergistic action when both BsAb are used in combination to target saporin against CD7 and CD38 expressed on the surface of the HSB-2 cell line.


Br. J. Cancer (1992), 65, 545–551 © Macmillan Press Ltd., 1992

### Effectiveness of combinations of bispecific antibodies for delivering saporin to human acute T-cell lymphoblastic leukaemia cell lines via CD7 and CD38 as cellular target molecules

D.J. Flavell[1], S. Cooper[1], B. Morland[2], R. French[3] & S.U. Flavell[1]

[1]*The Simon Flavell Leukaemia Research Laboratory, University Department of Pathology;* [2]*University Department of Child Health and* [3]*Tenovus Research Laboratory, Southampton General Hospital, Southampton SO9 4XY, UK.*

**Summary** We have investigated the effectiveness of three different $F(ab'\gamma)_2$ bispecific antibodies (BsAb) for delivering the ribosome inactivating protein (RIP) saporin via the CD7 or CD38 cell surface molecules to the human T-ALL cell lines HSB-2 and HPB-ALL. Inhibition of $^3$H-leucine uptake by target cells was used as the parameter of cellular cytotoxicity. Used singly against HSB-2 cells in the presence of varied concentrations of saporin, an anti-CD7 BsAb, (HB2 × DB7–18) and an anti-CD38 BsAb (OKT10 × RabSap), gave 435- and 286-fold increases in saporin toxicity, respectively. For HPB-ALL cells the anti-CD7 BsAb performed poorly giving only an eight-fold increase in toxicity whilst on the same cell line the anti-CD38 BsAb was highly potent giving an 80,000-fold increase in saporin toxicity.

A combination of both BsAb used together against HSB-2 cells was ten times more effective, than the best single BsAb HB2 × DB7–18 used alone. Kinetic studies conducted with HSB-2 cells revealed that the BsAb combination also gave an increased rate of protein synthesis inactivation in comparison to either BsAb used alone. These investigations clearly demonstrate a synergistic action when both BsAb are used in combination to target saporin against CD7 and CD38 expressed on the surface of the HSB-2 cell line.

The principle of using monoclonal antibodies for delivering drugs or toxins to distinct molecular structures expressed on the surface of unwanted tumour cells is an attractive one and has come under intense investigation in recent years (Hertler & Frankel, 1989). Theoretically, such a targeted approach to cancer therapy could offer a major advance in the selective elimination of tumour cells, improving the effective tumour cell kill rate whilst reducing the toxicity of treatment for non-target tissues overall. In practice many problems still need to be overcome before immunotoxin or antibody-drug conjugate-type therapies can be truly effective *in vivo* (Frankel *et al.*, 1986).

One potential limitation to the success of any targeted approach to therapy would be the heterogeneity of target antigen expression within a population of tumour cells. It follows that if a small number of cells within the tumour were negative for a single target antigen or, expressed the antigen only very weakly, then these cells would escape destruction due to a failure of antibody-mediated delivery of the cytotoxic agent to those particular cells. A possible means of overcoming this potential problem would be to target the cytotoxic agent against more than one cell surface target molecule, in the expectation that target tumour cells which were multiple antigen negative would occur with a lower frequency than single antigen negative target cells. This strategy would not only improve the likelihood of delivering the cytotoxic agent to all cells within the tumour, but would also ensure delivery of greater amounts of the agent to those cells that express all the target antigens. To investigate this possibility we have studied the cytotoxic efficacy of three different bispecific antibodies (BsAb) used singly or in combination, for delivering the ribosome inactivating protein (RIP) saporin to two different human T-ALL cell lines using CD7 and CD38 as the cellular target molecules.

### Materials and methods

### *Human acute T-cell leukaemia cell lines*

The T-ALL cell lines HSB-2 and HPB-ALL were used in these studies. Both cell lines were maintained in RPMI 1640 medium containing 10% foetal calf serum (Gibco), and supplemented with 1 mmol glutamine, 1 mmol sodium pyruvate, 100 IU $ml^{-1}$ benzyl penicillin and 100 $\mu g$ $ml^{-1}$ streptomycin sulphate. Cells were maintained continuously in the logarithmic phase of growth by passage at regular intervals.

### *Saporin*

The ribosome inactivating protein (RIP) saporin was purified from the seeds of *Saponaria officinalis* as described previously (Stirpe *et al.*, 1983).

### *Antibodies*

Ascitic fluids containing the anti-saporin mouse monoclonal antibody DB7–18, the anti-CD7 antibody HB2 and the anti-CD38 antibody OKT10, all murine antibodies of the IgG1 subclass were produced by injecting $1 \times 10^7$ of the appropriate hybridoma cell line into the peritoneal cavity of pristane primed BALB/c mice.

Polyclonal antisera reacting with saporin were raised in half lop rabbits using standard immunisation protocols (Glennie *et al.*, 1987).

The 7S IgG fractions of ascitic fluids and polyclonal antisera were isolated by precipitation with 2 M ammonium sulphate followed by ion exchange chromatography on Tris-acryl-M-DEAE (Elliott *et al.*, 1987). $F(ab')_2$ fragments from both monoclonal and polyclonal IgG were prepared by limited proteolysis with pepsin at pH 4.2 as described previously (Glennie *et al.*, 1987).

### *Construction of bispecific $F(ab')_2$ antibodies*

Heterodimeric $F(ab'\gamma)_2$ molecules containing two different mouse Fab' fragments or a mixture of mouse and rabbit Fab' fragments were constructed as described by Glennie *et al.* (1987). Briefly, $F(ab'\gamma)_2$ fragments of the required antibody were reduced to obtain Fab' fragments with hinge region SH groups, Fab'(SH). The SH groups on one of the Fab' (SH) species were then fully alkylated with excess O-phenylenedimaleimide to provide free maleimide groups. The two preparations, Fab'(mal) and Fab'(SH) were then combined under conditions which allowed cross linking of the maleimide and SH groups and avoided reoxidation of the SH groups. The final products were reduced and alkylated to

Correspondence: D.J. Flavell.
Received 12 July 1991; and in revised form 6 December 1991.

remove any minor untoward products which may have formed by oxidation or disulphide exchange and the final mixture fractionated according to size by chromatography on Ultragel AcA44 (LKB-Produkter AB, Bromma, Sweden). Three different F(ab'γ)$_2$ BsAb were constructed in this way the details of which are given in Table 1.

*Analysis of cell surface expression of CD7 and CD38 by flow cytometry*

Cells were analysed for surface expression of CD7 and CD38 by flow cytometry. Cells were incubated for 1 h at room temperature with a saturating concentration of HB2 (anti-CD7) or OKT10 (anti-CD38) antibody in the presence of 0.01% sodium azide. Following the first incubation cells were washed twice and incubated with a 1:20 dilution of fluorescein isothiocyanate conjugated F(ab')$_2$ fragments of a rabbit anti-mouse immunoglobulins antisera (Sigma Chemical Co., Poole, UK). Fluorescence intensity was then evaluated for 10,000 ungated cells on a FACSCAN flow cytometer.

*$^3$H-leucine uptake*

Protein synthesis levels in target cells exposed to BsAb and saporin were measured by $^3$H-leucine uptake. Triplicate cultures of HSB-2 or HPB-ALL cells at a density of $1 \times 10^5$ cells per well in 96 well microculture plates were exposed for 48 h at 37°C to BsAb and saporin at each experimental concentration. Each culture was then exposed for 12 h to 1 μCi $^3$H-leucine (TRK 510, Amersham International, Amersham, UK) and finally harvested onto glassfibre filters using a Skatron cell harvester. The amount of radioactive leucine incorporated by cells was measured by scintillation counting the harvested cells on the glassfibre discs in a Packard scintillation counter. Results obtained for experimental cultures are expressed as a percentage of the amount of $^3$H-leucine incorporation observed in untreated control cultures maintained under identical conditions.

*Kinetic studies*

The kinetics of protein synthesis inactivation brought about in HSB-2 cells by the anti-CD7 BsAb (HB2 × DB7-18) and the anti-CD38 BsAb (OKT10 × RabSap) when used singly or together in combination, was determined in 96 well microcultures of cells exposed to various concentrations of saporin (range $10^{-11}$ M to $10^{-7}$ M). In these investigations the BsAb HB2 × DB7-18 was used at a concentration of 0.1 μg ml$^{-1}$ and the BsAb OKT10 × Rabsap at 1 μg ml$^{-1}$. HSB-2 cells were incubated for 2 h in supplemented leucine-free RPMI medium at 37°C and then triplicate samples of $1 \times 10^5$ cells added to wells of a 96 well microculture plate containing each appropriate concentration of saporin and each BsAb singly or in combination, in supplemented leucine-free RPMI. Microculture plates were maintained at 37°C in a humidified atmosphere of 5% $CO_2$ in air and at 2, 6, 12, 24 and 48 h 1.0 μCi $^3$H-leucine was added to each well of the appropriate cultures and cells harvested after a 1 h pulse exposure onto glassfibre mats as described above. Regression analysis of $^3$H-leucine incorporation levels (expressed as a percentage of untreated control cultures) versus each time-point studied was undertaken for each saporin concentration employed. The time taken to reduce the protein synthesis level of HSB-2 cells by one log is defined as the $t_{10}$ and was obtained from the intercept point of the regression line with the 10% level on the regression chart.

### Results

*Cell surface expression of CD7 and CD38 by HSB-2 and HPB-ALL*

The expression of cell surface CD7 (MoAb HB2) and CD38 (MoAb OKT10) by HSB-2 and HPB-ALL cells was determined by flow cytometry and the profiles obtained for each cell line are shown in Figure 1. CD7 was expresssed strongly by 98% of HSB-2 cells with a mean fluorescent intensity of 259 arbitrary units (Figure 1a). In contrast 90% of HPB-

**Table 1** Specificities of F(ab'γ)$_2$ BsAbs used in this study

| *BsAb* | *Specificities* |
|---|---|
| HB2 × DB7-18 | anti-CD7/anti-saporin (all monoclonal)[a] |
| OKT10 × DB7-18 | anti-CD38/anti-saporin (all monoclonal)[a] |
| OKT10 × RabSap | anti-CD38/anti-saporin (mono/polyclonal)[b] |

[a]Constructed with two different monoclonal Fab' fragments. [b]Constructed with one monoclonal Fab' fragment (OKT10 anti-CD38) and rabbit anti-saporin Fab' fragments (RabSap).

**Figure 1** Flow cytometry profiles of CD7 and CD38 expression by HSB-2 and HPB-ALL cells. Broken line indicates negative control population, solid line indicates stained population.

ALL cells were effectively negative for CD7 expression with a subpopulation comprising approximately 10% of the total expressing with a mean fluorescent intensity of 54 (Figure 1b). HSB-2 cells expressed CD38 only relatively weakly with a mean fluorescent intensity for the whole population of only eight arbitrary units (Figure 1c). HPB-ALL cells expressed CD38 only moderately strongly with a mean fluorescent intensity of 90 arbitrary units (Figure 1d).

*Titration of each BsAb singly or in combination against HSB-2 cells in the presence of saporin*

To determine the optimum concentration of antibody required to achieve maximal protein synthesis inhibition, each BsAb was titrated against HSB-2 and HPB-ALL cells in the presence of a sub-toxic concentration of saporin (0.1 μg $ml^{-1} = 3.3 \times 10^{-9}$ M). Protein synthesis levels were evaluated after 48 h of continuous exposure and the results obtained for this titration are shown in Figure 2. The CD7 BsAb HB2 × DB7-18 performed best, achieving its $IC_{50}$ at an antibody concentration of 0.012 μg $ml^{-1}$ whilst the CD38 BsAb OKT10 × RabSap constructed with rabbit polyclonal anti-saporin Fab′ fragments reached its $IC_{50}$ at a concentration of 0.17 μg $ml^{-1}$. The CD38 BsAb OKT10 × DB7-18 constructed with mouse monoclonal anti-saporin Fab′ did not inhibit protein synthesis in HSB-2 cells over the entire range of BsAb concentrations investigated. Near maximal protein synthesis inactivation in HSB-2 cells occurred at BsAb concentrations of 0.1 μg $ml^{-1}$ for HB2 × DB7-18 and 1 μg $ml^{-1}$ for OKT10 × RabSap. The same optimal concentrations of BsAb were also demonstrated for the HPB-ALL cell line in identical experiments (data not shown). On the basis of these titration results obtained for single BsAb we elected to employ HB2 × DB7-18 at 0.1 μg $ml^{-1}$ and OKT-10 × RabSap at 1 μg $ml^{-1}$ throughout these studies.

When a 1 to 10 ratio mixture of the CD7 and CD38 BsAb HB2 × DB7-18 and OKT10 × RabSap was titrated against HSB-2 cells, the combination of both BsAb performed better than either BsAb used alone, the combination giving an $IC_{50}$ of 0.008 μg $ml^{-1}$ (this value being an expression of the concentration of HB2 × DB7-18 BsAb in the mixture). Each BsAb used in the absence of saporin over the range of concentrations investigated had no significant effect on HSB-2 protein synthesis levels (for the sake of graphical clarity data for the BsAb HB2 × DB7-18 only is shown in Figure 2). Similarly, equimolar mixtures of pairs of relevant $F(ab'\gamma)_2$ fragments from which each BsAb was constructed used over the same concentration range, together with saporin at 0.1 μg $ml^{-1}$ ($3.3 \times 10^{-9}$ M), had no significant effect on HSB-2 protein synthesis levels (data for only HB2 + DB7-18 is shown in Figure 2).

*Titration of saporin against HSB-2 and HPB-ALL cells in the presence of single or combinations of BsAb*

Cultures of HSB-2 or HPB-ALL cells were exposed to saporin alone ranging in concentration from $10^{-13}$ M to $10^{-6}$ M or together with the CD7 BsAb HB2 × DB7-18 (0.1 μg $ml^{-1}$), the CD38 BsAb OKT10 × RabSap (1 μg $ml^{-1}$) or a combination of both BsAb together (0.1 μg $ml^{-1}$ HB2 × DB7-18 + 1 μg $ml^{-1}$ OKT10 × RabSap). HSB-2 and HPB-ALL cells were also exposed to the monoclonal CD38 BsAb OKT10 × DB7-18 (1 μg $ml^{-1}$) singly or in combination with the CD7 BsAb HB2 × DB7-18 together with saporin over the range of saporin concentrations. Cells were also exposed to saporin at the various concentrations together with equivalent amounts of an eqimolar mixture of pairs of the relevant $F(ab'\gamma)_2$ fragments from which each BsAb was constructed. The protein synthesis levels, expressed as a percentage of control levels, in cultures of cells continuously exposed for 48 h to each treatment are shown graphically in Figures 3a (HSB-2 cells) and 3b (HPB-ALL cells) and the $IC_{50}$ and fold increase values summarised in Table II.

**Figure 2** [3]H-leucine incorporation levels in HSB-2 cells exposed to various concentrations of the BsAb's HB2 × DB7-18 (▲——▲), OKT10 × RabSap (●——●), a combination of both these BsAb's together or to the BsAb OKT10 × DB7-18 (O---O) in the presence of saporin at 0.1 μg $ml^{-1}$. Levels are shown for HSB-2 cells exposed to the BsAb HB2 × DB7-18 without saporin (□---□). Bars indicate one standard deviation.

**Figure 3** [3]H-leucine incorporation levels in **a**, HSB-2 or **b**, HPB-ALL cells exposed to various concentrations of saporin alone (■——■) or together with 0.1 μg $ml^{-1}$ HB2 × DB7-18 BsAb (CD7) (▲——▲), 1 μg $ml^{-1}$ OKT10 × RabSap BsAb (CD38) (●——●), a combination of both BsAb's together (CD7-+ CD38) (∇---∇) or an equimolar mixture of HB2 + DB7-18 $F(ab'\gamma)_2$ fragments (□---□). Bars indicate one standard deviation.

**Table 2** Inhibition of protein synthesis in HSB-2 and HPB-ALL cells by saporin delivering bispecific antibodies used singly or in combination

| *BsAb* | $^{a}IC_{50}$ *HSB-2* | *HPB-ALL* | *Fold increase*[b] *HSB-2* | *HPB-ALL* |
|---|---|---|---|---|
| HB2 × DB7–18 (CD7) | 0.23 nmol | 5.00 nmol | 435 | 8 |
| OKT10 × RabSap (CD38) | 0.35 nmol | 0.50 pmol | 286 | 80,000 |
| OKT10 × DB7–18 (CD38) | 5.00 nmol | 2.00 nmol | 20 | 20 |
| HB2 × DB7–18 + OKT10 × RabSap | 0.025 nmol | 0.80 pmol | 4,000 | 50,000 |
| HB2 × DB7–18 + OKT10 × DB7–18 | 0.50 nmol | 1.50 nmol | 200 | 27 |
| Saporin only | 100.00 nmol | 40.00 nmol | | |

[a]$IC_{50}$ calculated as the concentration of saporin needed to give 50% inhibition of $^{3}$H-leucine incorporation compared with untreated control cells. [b]Fold increase calculated as $IC_{50}$ saporin alone/$IC_{50}$ of BsAb + saporin.

The results obtained for HSB-2 cells are shown in Figure 3a. The $IC_{50}$ for saporin alone on HSB-2 cells was found to be 100 nmol. The CD7 BsAb, HB2 × DB7–18 increased the toxicity of saporin for HSB-2 cells 435-fold, decreasing the $IC_{50}$ to 0.23 nmol whilst the CD38 BsAb OKT10 × RabSap increased toxicity 286-fold with a decrease in the $IC_{50}$ to 0.35 nmol. When both of these BsAb were used in combination an $IC_{50}$ value of 0.025 nmol was obtained, this representing a 4,000-fold increase in saporin toxicity. Thus, both BsAb used in combination exerted more than just an additive effect. Neither pair of $F(ab'\gamma)_2$ fragments used together with saporin at the various concentrations had any significant influence on protein synthesis levels. For the sake of graphical clarity only the curve for HB2 + DB7–18 $F(ab'\gamma)_2$ fragments is shown in Figure 3a. Examination of the curves in Figure 3a shows that at concentrations of saporin between $10^{-12}$ M and $10^{-10}$ M the CD38 BsAb OKT10 × RabSap performed better than the CD7 BsAb HB2 × DB7–18, but performance began to decline at concentrations of saporin above $10^{-10}$ M. In comparison the CD7 BsAb continued to perform well up to $10^{-9}$ M, and outperformed the CD38 BsAb at saporin concentrations above $1.5 \times 10^{-10}$ M, the point at which the two curves cross over on the graph (Figure 3a). The monoclonal CD38 BsAb OKT10 × DB7–18 used at 1 $\mu g\ ml^{-1}$ performed poorly against HSB-2 cells giving an $IC_{50}$ of 5 nmol, this representing only a 20-fold increase in saporin toxicity (Table 2).

The results obtained on HPB-ALL cells are shown in Figure 3b. The $IC_{50}$ for saporin alone was found to be 40 nmol, revealing this cell line to be two and a half times more sensitive to saporin than HSB-2. The CD7 BsAb HB2 × DB7–18 increased saporin toxicity only eight-fold decreasing the $IC_{50}$ to 5 nmol. The CD38 BsAb OKT-10 × RabSap was highly effective, increasing saporin toxicity 80,000-fold with a reduction in the $IC_{50}$ to 0.5 pmol. In contrast the monoclonal CD38 BsAb OKT10 × DB7–18 performed poorly against HPB-ALL cells giving an $IC_{50}$ of 2 nmol this representing only a twenty-fold increase in saporin toxicity (Table 2). Interestingly, when both the anti-CD7 BsAb HB2 × DB7–18 and anti-CD38 BsAb OKT-10 × RabSap were used in combination against HPB--ALL cells, they were somewhat less effective than OKT10 × RabSap used alone, the combination giving only a 50,000-fold increase in saporin toxicity compared with the 80,000-fold increase obtained for OKT10 × RabSap alone.

### *Kinetics of protein synthesis inactivation*

Experiments were conducted to determine the rate at which various concentrations of saporin inactivated protein synthesis in HSB-2 cells in the presence of each BsAb used either singly or in combination. In these experiments the concentration of BsAb was kept constant at 0.1 $\mu g\ ml^{-1}$ for HB2 × DB7–18 or 1.0 $\mu g\ ml^{-1}$ for OKT10 × RabSap. The rate slopes expressed as a percentage of the control level of $^{3}$H-leucine incorporation with respect to time, obtained for HSB-2 cells treated with individual or a combination of both BsAbs are shown in Figure 4. The rate of inactivation was clearly shown to be concentration dependent and linear. The time taken for 90% inhibition of protein synthesis relative to an equivalent number of untreated control cells is defined as the $t_{10}$ and this value plotted against each concentration of saporin used in the presence of each BsAb singly or in combination is shown in Figure 5. Of the two BsAb, the CD7 BsAb HB2 × DB7–18 clearly gave the most rapid rate of protein synthesis inactivation for concentrations of saporin between $10^{-7}$ M and $10^{-9}$ M. Interestingly, at a saporin concentration of $10^{-10}$ M the CD38 BsAb OKT10 × RabSap inactivated protein synthesis more rapidly with a $t_{10}$ of 88 h compared with 226 h for the CD7 BsAb. When the CD7 and CD38 BsAb were used in combination the rate of protein synthesis inactivation was substantially increased over the entire range of saporin concentrations used (Figure 5). Thus, at a saporin concentration of $10^{-10}$ M the $t_{10}$ obtained for the two BsAb used in combination was 48 h compared with 88 h for the CD38 BsAb and 226 h for the CD7 BsAb when used alone. At a saporin concentration of $10^{-11}$ M the individual CD7 and CD38 BsAb were both ineffective. In contrast the combination of both BsAb together, with saporin at $10^{-11}$ M was effective giving a $t_{10}$ value of 85 h.

### Discussion

The studies described here have clearly demonstrated that the effective cytotoxic dose of saporin that is delivered to HSB-2 cells is improved approximately ten-fold when targeted against both CD7 and CD38 in comparison to either target molecule alone. The CD7 BsAb HB2 × DB7–18 has proven highly effective at selectively delivering saporin to HSB-2 cells in this and previous studies (Flavell *et al.*, 1991), there being a clearly demonstrable dose response effect obtained when either BsAb or saporin concentration are taken into account. In the experiments described in the present paper, the CD38 BsAb OKT10 × RabSap, constructed with Fab′ fragments from a rabbit anti-saporin polyclonal antisera was also effective at delivering a cytotoxic dose of saporin to HSB-2 cells and again there was a clearly demonstrable dose response effect obtained when either BsAb or saporin concentration were taken into consideration. The exquisite specificity with which saporin is delivered to only the target cell via the CD38 molecule has also been unequivocally demonstrated for the BsAb OKT10 × RabSap. Thus, this BsAb fails to deliver saporin to the $CD38^{-}$ cell line HL60 and moreover, its ability to deliver a cytotoxic dose of saporin to HSB-2 cells is abrogated in the presence of a ten-fold excess of free OKT10 (CD38) antibody (data not shown). In the context of specificity we have also demonstrated that an irrelevant BsAb

**Figure 4** Kinetics of protein synthesis inactivation in HSB-2 cells exposed to a range of saporin concentrations ($10^{-11}$ M to $10^{-7}$ M) together with **a**, HB2 × DB7-18 0.1 μg ml$^{-1}$, **b**, OKT10 × RabSap 1 μg ml$^{-1}$ or **c**, a combination of both BsAb's used together. Panel **d**, shows the inactivation rate in HSB-2 cells exposed to the various concentrations of saporin alone. Regression coefficients obtained at each saporin concentration for each treatment were as follows: **a**, HB2 × DB7-18 (0.1 μg ml$^{-1}$) r = 0.983 ($10^{-7}$ M), r = 0.999 ($10^{-8}$ M), r = 0.992 ($10^{-9}$ M), r = 0.567 ($10^{-10}$ M), r = 0.610 ($10^{-11}$ M **b**, OKT10 × RabSac (1 μg ml$^{-1}$) r = 0.957 ($10^{-7}$ M), r = 0.932 ($10^{-8}$ M), r = 0.953 ($10^{-9}$ M), r = 0.907 ($10^{-10}$ M), r = 0.094 ($10^{-11}$ M) **c**, HB2 × DB7-18 + OKT10 × RabSap COMBINATION r = 0.984 ($10^{-7}$ M), r = 0.955 ($10^{-8}$ M), r = 0.988 ($10^{-9}$ M), r = 0.970 ($10^{-10}$ M), r = 0.982 ($10^{-11}$ M).

RJD × DB7-18 with target cell specificity for an idiotypic determinant on a mouse B-cell leukaemia cell line in one arm and saporin in the other, had no influence on the protein synthesis inhibitory effects of saporin on either HSB-2 or HPB-ALL (data not shown).

On HSB-2 cells the CD38 BsAb OKT10 × RabSap gave an

**Figure 5** Kinetics of protein synthesis inactivation in HSB-2 cells. The $t_{10}$ values (time in hours to reduce protein synthesis levels in treated cells to 10% of that seen in untreated control cells) were obtained from the rate slopes shown in Figure 4 following exposure of HSB-2 cells to a range of saporin concentrations ($10^{-11}$ M to $10^{-7}$ M), together with the BsAb HB2 × DB7-18 (■——■), OKT10 × RabSap (▲——▲) or a combination of both BsAb's (●——●).

observed 286-fold increase in saporin toxicity compared with the 435-fold increase observed for the CD7 BsAb HB2 × DB7-18. The monoclonal CD38 BsAb OKT10-× DB7-18 performed poorly on HSB-2 cells giving only a twenty-fold increase in saporin toxicity.

When the BsAb HB2 × DB7-18 and OKT10 × RabSap were used in combination against HSB-2 cells their effectiveness was improved almost ten-fold compared with the best BsAb, HB2 × DB7-18 used alone, the combination giving a 4,000-fold increase in saporin toxicity. Thus, the effect of both BsAb used in combination was more than just additive and this probably reflects the fact that greater numbers of toxin molecules gain entry to the cytosol with a subsequent increase in the probability of achieving a hit on target ribosomes. In the presence of the combination of BsAb there is also the possibility of both derivatives binding to a single saporin molecule due to the recognition of different saporin epitopes. Such bivalent binding would allow cross linking of adjacent CD7 and CD38 molecules on the target cell surface and this may favour saporin internalisation and/or availability. When a combination of the CD7 BsAb HB2 × DB7-18 was used with the monoclonal CD38 BsAb OKT10 × DB7-18 the mixture actually performed less well than the CD7 BsAb alone. This can probably be accounted for by a reduction in the amount of saporin available for delivery to the target cell by the highly effective BsAb HB2 × DB7-18 due to competition for free saporin by the relatively ineffective BsAb OKT10 × DB7-18.

In addition to an improved cytotoxicity, the combination of BsAb also gave a substantially faster rate of protein synthesis inactivation in HSB-2 cells. Similar increases in the cytotoxic efficacy and rate of protein synthesis inactivation have been reported for combinations of IT's for targeting intact ricin against the CD2, CD5, CD7, and CD18 molecules on the surface of the T-ALL cell line CEM (Strong *et al.*, 1985). It is our opinion that this increase may be explained as a purely quantitative phenomenon and probably does not represent a real increase in the rate of BsAb-saporin internalisation or translocation from the endosome to cytosol. Evidence in support of this contention comes from our preliminary investigations which demonstrate that the modulation rate for each BsAb is unaltered when the two BsAb are used in combination (Morland, unpublished results). We feel that it is much more likely that the higher concentration of endosomal saporin achieved when this RIP is delivered to two different molecules on the cell surface results in more saporin gaining access to the cytosol per unit time simply because more molecules are available for translocation. It is currently not known whether saporin simultaneously targeted against both CD7 and CD38 on the same cell surface enters the cell using a common endocytic vesicle or whether each enters via a different route. Carriere *et al.* (1989) demonstrated that internalization of a CD7 MoAb by the T-ALL cell line CEM was via coated pits on the cell membrane. In contrast internalisation of a CD5 MoAb rarely occurred via these structures and for a CD4 MoAb it never occurred.

The CD38 BsAb OKT10 × RabSap constructed with rabbit polyclonal anti-saporin Fab' fragments was significantly more potent against HPB-ALL than HSB-2 cells, giving an 80,000-fold increase in saporin toxicity ($IC_{50}$ 0.5 pmol). In contrast the CD7 BsAb HB2 × DB7-18 performed poorly against HPB-ALL giving only an eight-fold increase in toxicity. This poor performance probably reflects the low level of expression of CD7 by HPB-ALL and the subsequent poor delivery of saporin to the target cell surface. The combination of both HB2 × DB7-18 and OKT10 × RabSap performed less well ($IC_{50}$ 0.8 pmol 50,000-fold) than OKT10 × RabSap used alone ($IC_{50}$ 0.5 pmol 80,000-fold). Again, this probably reflects competition for saporin by the relatively ineffective (for HPB-ALL) BsAb HB2 × DB7-18, thereby reducing the effective amount of saporin available for delivery to the target cell by the highly potent BsAb OKT10 × RabSap. This type of effect resulting in decreased efficacy when one of the target molecules is expressed minimally on the target cell would not occur with conventional immunotoxins and we are currently conducting studies to explore this matter further.

The poor performance of the monoclonal CD38 BsAb OKT10 × DB7-18 (constructed with a monoclonal anti-saporin Fab') when tested on either HSB-2 or HPB-ALL, may indicate that cross linking of adjacent saporin molecules bound at the target cell surface may be required for the effective delivery of saporin to the cell interior via this particular cell surface molecule. Cross linking cannot be achieved by the BsAb OKT10 × DB7-18 which recognises only a single epitope on the saporin molecule and the failure of OKT10 × DB7-18 may therefore be due to the inability of this BsAb to cross link saporin molecules at the cell surface. In contrast the BsAb OKT10 × RabSap constructed with rabbit polyclonal anti-saporin Fab' fragments will recognise more than one saporin epitope and is therefore capable of cross linking adjacent saporin molecules at the cell surface. In this context French *et al.* (1991) have demonstrated that a BsAb constructed with a rabbit anti-saporin antiserum was more effective for targeting saporin against the guinea pig acute lymphoblastic leukaemia cell line $L_2C$ than BsAb constructed with a single monoclonal anti-saporin Fab arm. Combinations of BsAb constructed with different anti-saporin monoclonal Fab' fragments recognising different epitopes, performed considerably better than the single reagents alone. These workers suggested that the increased effective antibody affinity achieved for binding saporin to the target cell surface via two different saporin epitopes might account for the increased cytotoxicity.

One of the most important factors in determining the effectiveness of any method employed for targeting drugs or toxins to tumour cells remains the nature of the target molecule and its level of expression on the tumour cell surface. In order to be certain that drug or toxin is delivered to each and every cell would require that the target molecule be expressed ubiquitously by all cells within the tumour. Tumour cells are by their very nature heterogeneous in many respects, not least of all in their expression of cell surface molecules. It is clear that should just a few tumour cells show reduced or failed expression of the target molecule on the membrane surface, then effective amounts of the therapeutic agent would not be delivered and tumour regrowth would be likely to occur from these escapee cells. Such has been shown to be the case with the guinea pig acute lymphoblastic leukaemia cell line $L_2C$. Here Glennie *et al.* (1988) demonstrated that the $L_2C$ tumour cell population re-emerging following immunotherapy of tumour bearing animals with saporin and a BsAb targeting against an idiotypic determinant on the $L_2C$ cell surface in one Fab arm and saporin in the other arm, were target antigen negative. It was hypothesized that selective pressure put on the tumour cell population allowed for the emergence of an antigen negative subpopulation. Previous work of our own has shown that HSB-2 cells escaping destruction, following *in vitro* treatment with a BsAb targeting saporin against the CD7 molecule expressed on the surface of this cell line, express CD7 at the same level as untreated parent cells (Flavell *et al.*, 1991). This observation demonstrates that we have not selected for a truly antigen negative population and we have concluded that an epigenetic downregulation of CD7 by a minute subpopulation of HSB-2 cells allowed for their survival following their initial treatment with BsAb and saporin. Upon regrowth of the $CD7^-$ surviving population, CD7 expression was upregulated once again. On the basis of these two independent observations we are of the opinion that downregulation of target antigen expression due either to reversible epigenetic or an irreversible mutational event may both on different occasions be independently responsible for target cell escape.

In conclusion we have clearly demonstrated that targeting saporin against both CD7 and CD38 on the leukaemia cell surface increases cytotoxicity in more than just an additive fashion and moreover increases the rate at which protein synthesis is inactivated in the target cell. These findings are likely to be of some importance in the clinical utility of such therapeutic reagents in human leukaemia for a number of reasons. Firstly, multiple antigen targeting should contribute to overcoming problems encountered with single antigen negative tumour cells present within the population which would otherwise evade toxin delivery. Secondly, targeting against more than one cell surface molecule delivers greater quantities of saporin to those cells that express all target molecules and leads to a more rapid and more effective protein synthesis inactivation in the target cell. This is of obvious importance where the *in vitro* serum half life of BsAb or RIP is limited and where a more rapid rate of intoxication would be of positive value. *In vivo* therapy studies to investigate these matters employing severe combined immune deficient (scid) mice bearing human leukaemia xenografts are currently in progress.

We would like to thank Dr Martin Glennie and Ms Alison Tutt (Tenovus Laboratory, Southampton) for constructing BsAb. We would also like to thank Professor Fiorenzo Stirpe, University of Bolgona for kindly supplying saporin. This work was supported in part by Leukaemia Busters and Lederle Cyanamid, Gosport, UK. DJF and SUF are Cancer Research Campaign Research Fellows.

### References

CARRIERE, D., ARCIER, J.M., DEROCQ, J.M., FONTAINE, C. & RICHER, G. (1989). Antigenic modulation induced by four monoclonal antibodies absorbed on gold particles (specificity anti-CD4, anti-CD5, anti-CD7, and anti-150-kDa antigen): relationship between modulation and cytotoxic activity of immunotoxins. *Exp. Cell Res.*, **182**, 114.

ELLIOTT, T.J., GLENNIE, M.J., MCBRIDE, H.M. & STEVENSON, G.T. (1987). Analysis of the interaction of antibodies with immunoglobulin idiotypes on neoplastic B lymphocytes: implications for immunotherapy. *J. Immunol.*, **138**, 981.

FLAVELL, D.J., COOPER, S., MORLAND, B. & FLAVELL, S.U. (1991). Characteristics and performance of a bispecific $F(ab'\gamma)_2$ antibody for delivering saporin to a $CD7^+$ human acute T-cell leukaemia cell line. *Br. J. Cancer*, **63**, in press.

FRANKEL, A.E., HOUSTON, L.L. & ISSELL, B.F. (1986). Prospects for immunotoxin therapy in cancer. *Ann. Rev. Med.*, **37**, 125.

FRENCH, R.R., COURTENAY, A.E., INGAMELLIS, S., STIRPE, F., STEVENSON, G.T. & GLENNIE, M.J. (1991). Cooperative mixtures of bispecific $F(ab')_2$ antibodies for delivering saporin to lymphoma *in vitro* and *in vivo*. *Cancer Res.*, (in press).

GLENNIE, M.J., MCBRIDE, H.M., WORTH, A.T. & STEVENSON, G.T. (1987). Preparation and performance of bispecific $F(ab')_2$ antibody containing thioether-linked Fab' fragments. *J. Immunol.*, **139,** 2367.

GLENNIE, M.J., BRENNAND, D.M,. BRYDEN, F. & 4 others (1988). Bispecific $F(ab')_2$ antibody for the delivery of saporin in the treatment of lymphoma. *J. Immunol.*, **141,** 3662.

HERTLER, A.A. & FRANKEL, A.E. (1989). Immunotoxins: a clinical review of their use in the treatment of malignancies. *J. Clin. Oncol.*, **17,** 1932.

STIRPE, F., GASPERI-CAMPANI, G., BARBIERI, L., FALASCA, A., ABBONDANZA, A. & STEVENS, W.A. (1983). Ribosome-inactivating proteins from the seeds of *Saponaria officinalis L.* (soapwort), of *Agrostemma githago L.* (corn cockle) and of *Asparagus officinalis* (asparagus) and from the latex of *Hura crepitans L.* (sandbox tree). *Biochem. J.*, **216,** 617.

STRONG, R.C., UCKUN, F., YOULE, R.J., KERSEY, J.H. & VALLERA, D.A. (1985). Use of multiple T-cell directed intact ricin immunotoxins for autologous bone marrow transplantation. *Blood*, **66,** 627.